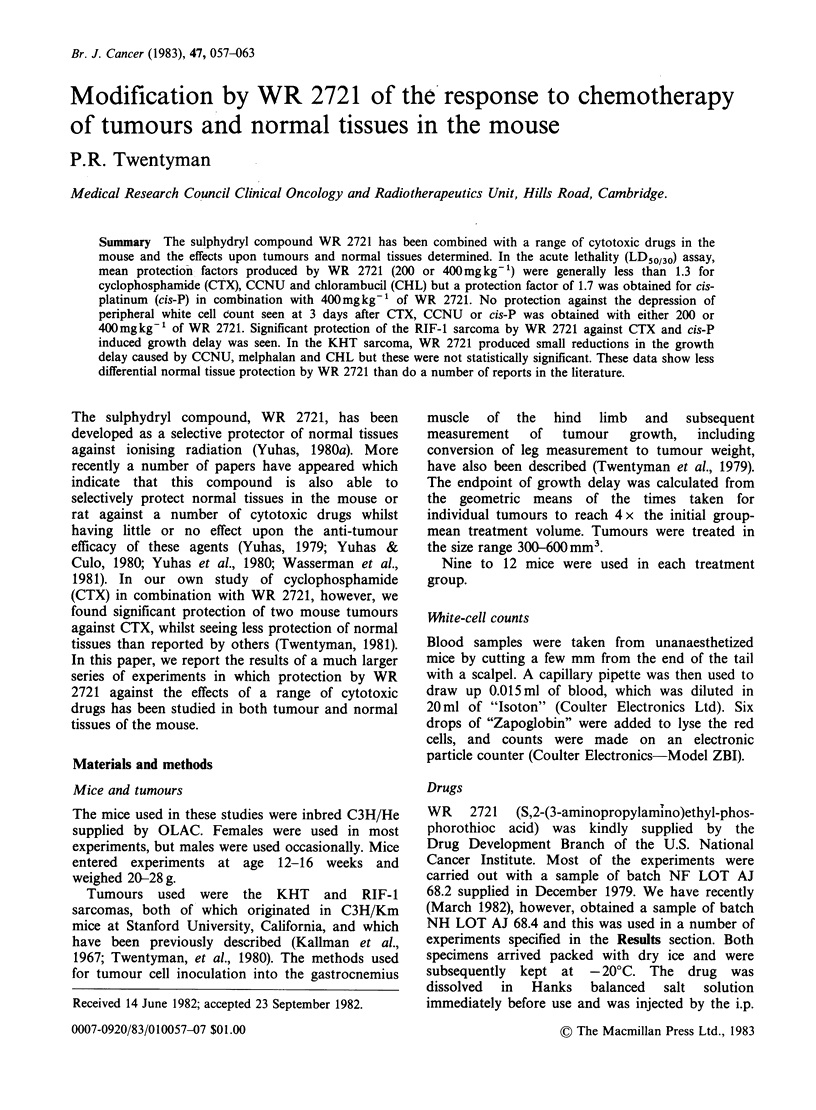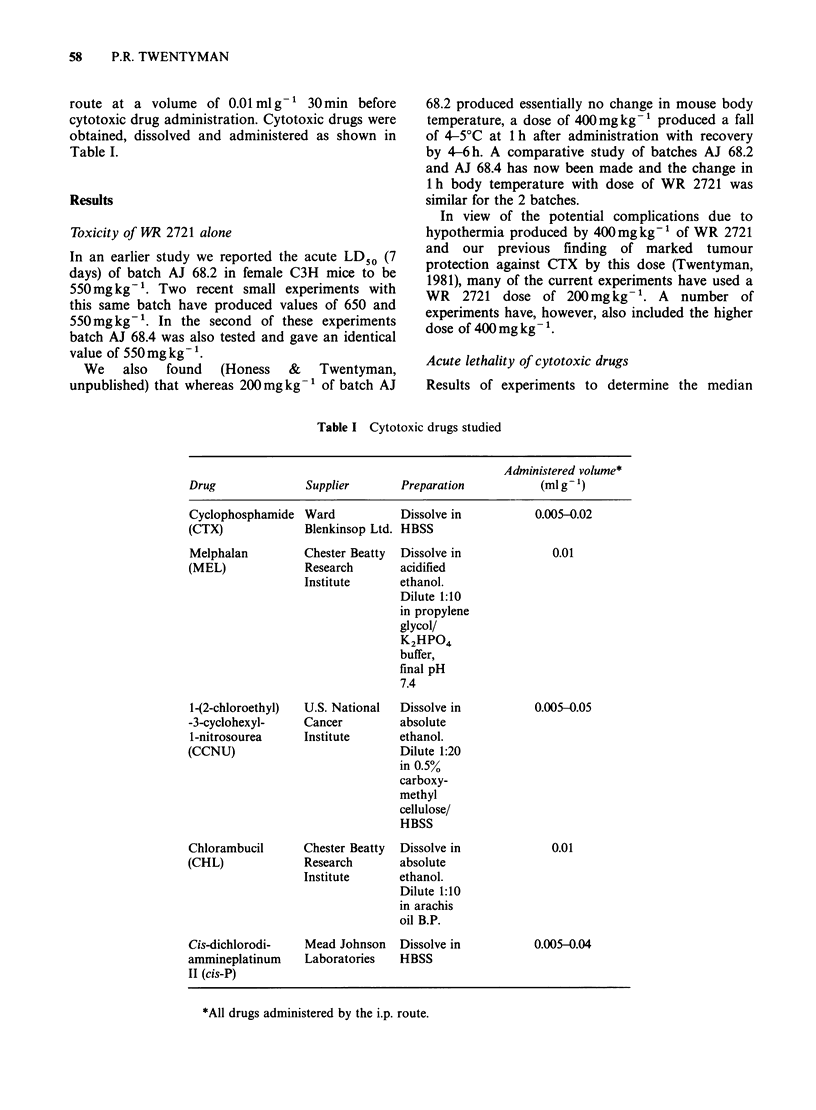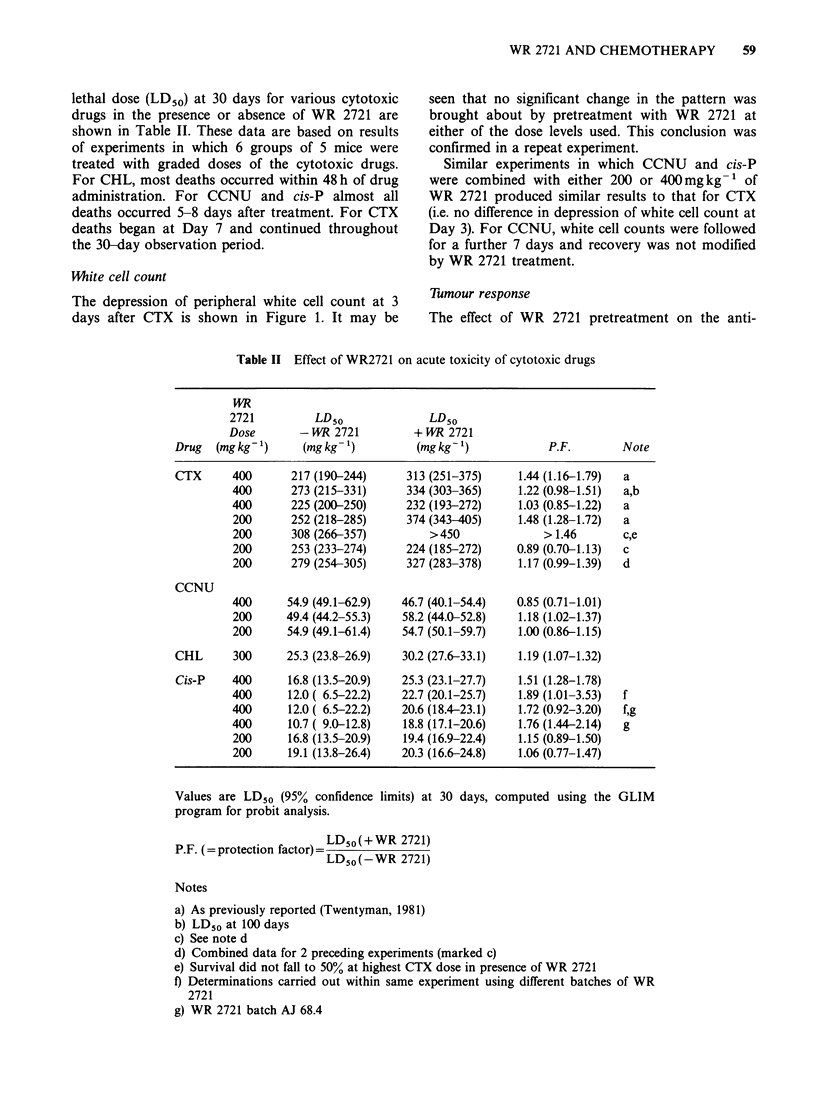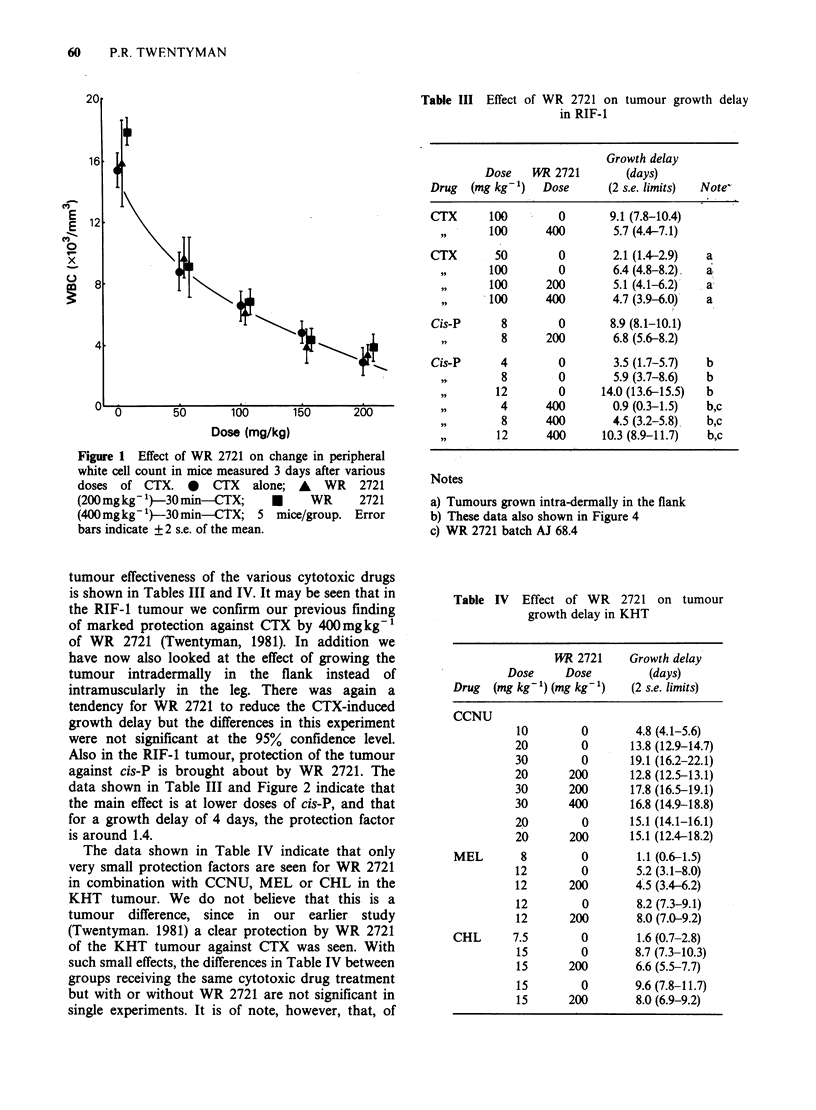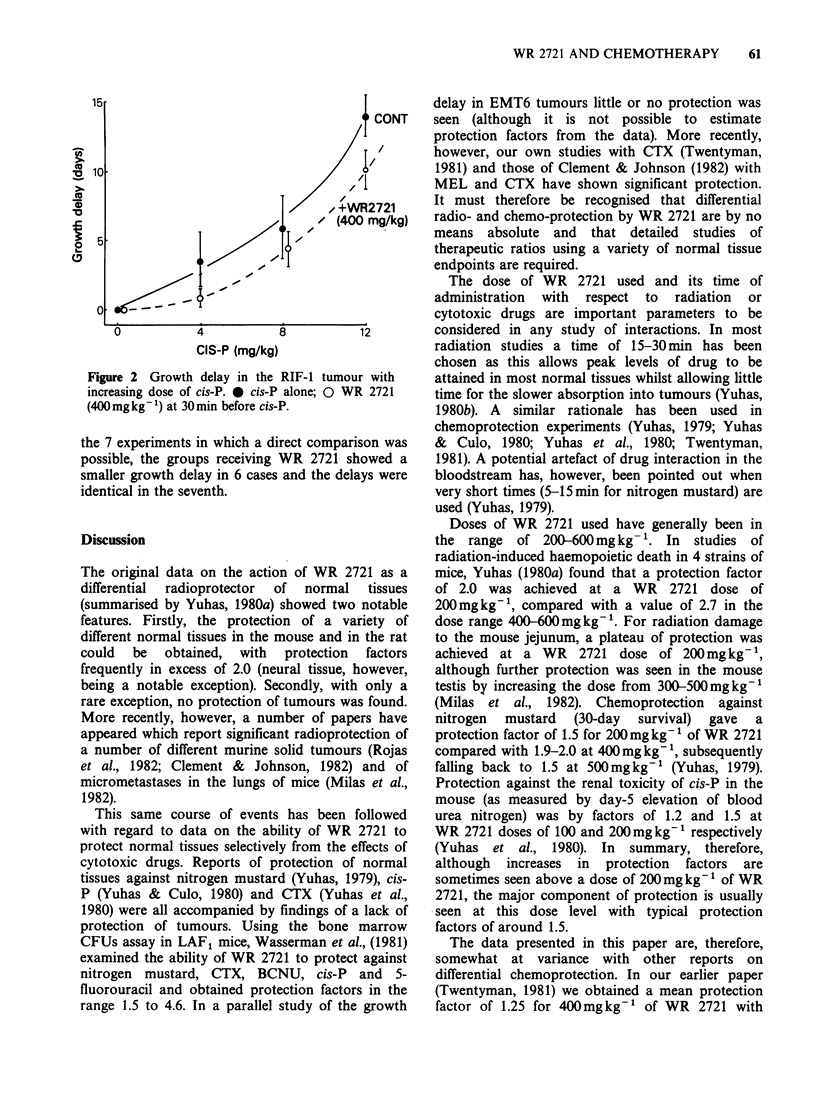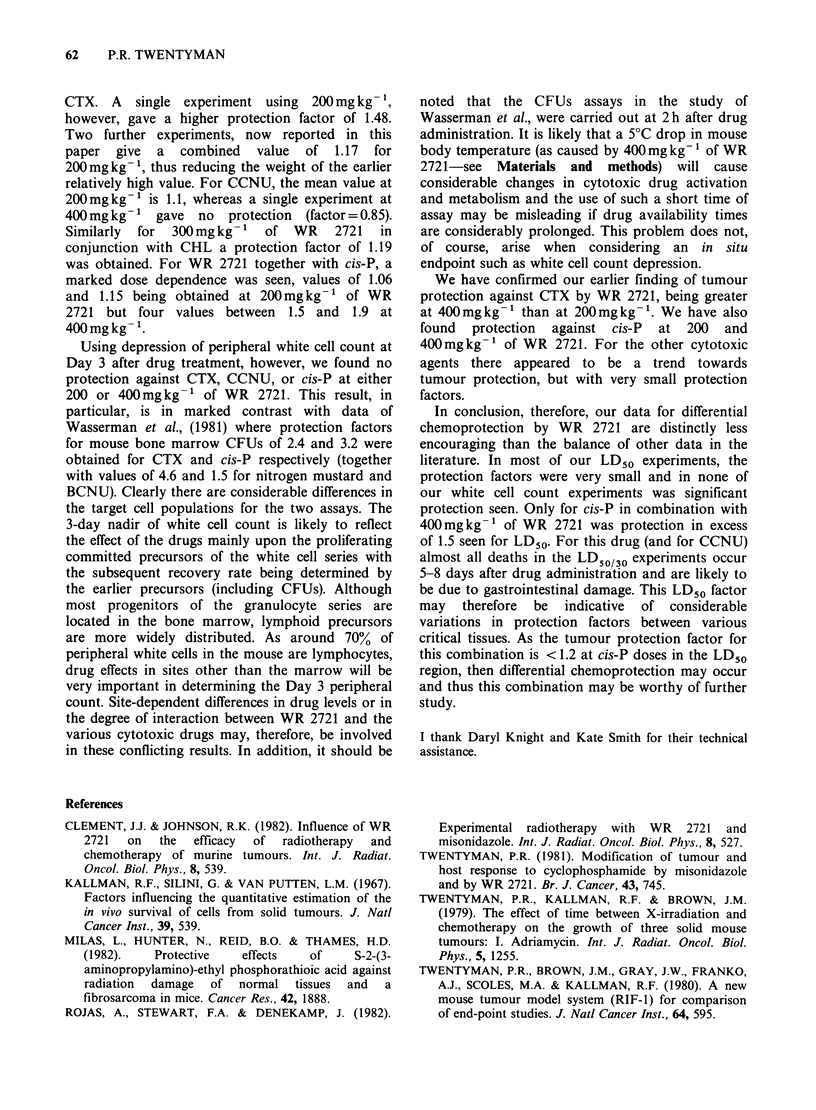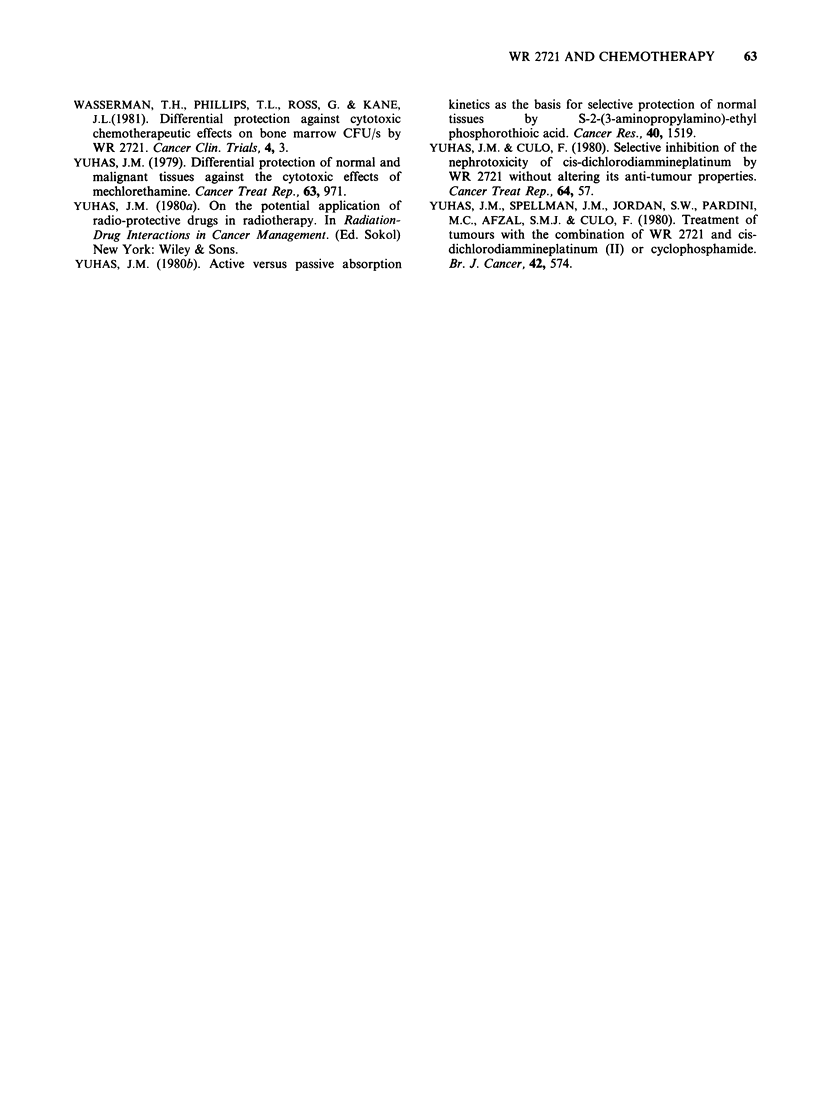# Modification by WR 2721 of the response to chemotherapy of tumours and normal tissues in the mouse.

**DOI:** 10.1038/bjc.1983.7

**Published:** 1983-01

**Authors:** P. R. Twentyman

## Abstract

The sulphydryl compound WR 2721 has been combined with a range of cytotoxic drugs in the mouse and the effects upon tumours and normal tissues determined. In the acute lethality (LD50/30) assay, mean protection factors produced by WR 2721 (200 or 400 mg kg-1) were generally less than 1.3 for cyclophosphamide (CTX), CCNU and chlorambucil (CHL) but a protection factor of 1.7 was obtained for cisplatinum (cis-P) in combination with 400 mg kg-1 of WR 2721. No protection against the depression of peripheral white cell count seen at 3 days after CTX, CCNU or cis-P was obtained with either 200 or 400 mg kg-1 of WR 2721. Significant protection of the RIF-1 sarcoma by WR 2721 against CTX and cis-P induced growth delay was seen. In the KHT sarcoma, WR 2721 produced small reductions in the growth delay caused by CCNU, melphalan and CHL but these were not statistically significant. These data show less differential normal tissue protection by WR 2721 than do a number of reports in the literature.


*Br. J. Cancer* (1983), **47**, 057–063

### Modification by WR 2721 of the response to chemotherapy of tumours and normal tissues in the mouse

P.R. Twentyman

*Medical Research Council Clinical Oncology and Radiotherapeutics Unit, Hills Road, Cambridge.*

**Summary** The sulphydryl compound WR 2721 has been combined with a range of cytotoxic drugs in the mouse and the effects upon tumours and normal tissues determined. In the acute lethality ($LD_{50/30}$) assay, mean protection factors produced by WR 2721 (200 or 400 mg kg$^{-1}$) were generally less than 1.3 for cyclophosphamide (CTX), CCNU and chlorambucil (CHL) but a protection factor of 1.7 was obtained for *cis*-platinum (*cis*-P) in combination with 400 mg kg$^{-1}$ of WR 2721. No protection against the depression of peripheral white cell count seen at 3 days after CTX, CCNU or *cis*-P was obtained with either 200 or 400 mg kg$^{-1}$ of WR 2721. Significant protection of the RIF-1 sarcoma by WR 2721 against CTX and *cis*-P induced growth delay was seen. In the KHT sarcoma, WR 2721 produced small reductions in the growth delay caused by CCNU, melphalan and CHL but these were not statistically significant. These data show less differential normal tissue protection by WR 2721 than do a number of reports in the literature.

The sulphydryl compound, WR 2721, has been developed as a selective protector of normal tissues against ionising radiation (Yuhas, 1980*a*). More recently a number of papers have appeared which indicate that this compound is also able to selectively protect normal tissues in the mouse or rat against a number of cytotoxic drugs whilst having little or no effect upon the anti-tumour efficacy of these agents (Yuhas, 1979; Yuhas & Culo, 1980; Yuhas *et al.*, 1980; Wasserman *et al.*, 1981). In our own study of cyclophosphamide (CTX) in combination with WR 2721, however, we found significant protection of two mouse tumours against CTX, whilst seeing less protection of normal tissues than reported by others (Twentyman, 1981). In this paper, we report the results of a much larger series of experiments in which protection by WR 2721 against the effects of a range of cytotoxic drugs has been studied in both tumour and normal tissues of the mouse.

### Materials and methods

### *Mice and tumours*

The mice used in these studies were inbred C3H/He supplied by OLAC. Females were used in most experiments, but males were used occasionally. Mice entered experiments at age 12–16 weeks and weighed 20–28 g.

Tumours used were the KHT and RIF-1 sarcomas, both of which originated in C3H/Km mice at Stanford University, California, and which have been previously described (Kallman *et al.*, 1967; Twentyman, *et al.*, 1980). The methods used for tumour cell inoculation into the gastrocnemius muscle of the hind limb and subsequent measurement of tumour growth, including conversion of leg measurement to tumour weight, have also been described (Twentyman *et al.*, 1979). The endpoint of growth delay was calculated from the geometric means of the times taken for individual tumours to reach 4× the initial group-mean treatment volume. Tumours were treated in the size range 300–600 mm$^3$.

Nine to 12 mice were used in each treatment group.

### *White-cell counts*

Blood samples were taken from unanaesthetized mice by cutting a few mm from the end of the tail with a scalpel. A capillary pipette was then used to draw up 0.015 ml of blood, which was diluted in 20 ml of "Isoton" (Coulter Electronics Ltd). Six drops of "Zapoglobin" were added to lyse the red cells, and counts were made on an electronic particle counter (Coulter Electronics—Model ZBI).

### *Drugs*

WR 2721 (S,2-(3-aminopropylamino)ethyl-phosphorothioc acid) was kindly supplied by the Drug Development Branch of the U.S. National Cancer Institute. Most of the experiments were carried out with a sample of batch NF LOT AJ 68.2 supplied in December 1979. We have recently (March 1982), however, obtained a sample of batch NH LOT AJ 68.4 and this was used in a number of experiments specified in the **Results** section. Both specimens arrived packed with dry ice and were subsequently kept at −20°C. The drug was dissolved in Hanks balanced salt solution immediately before use and was injected by the i.p.

Received 14 June 1982; accepted 23 September 1982.

0007-0920/83/010057–07 $01.00

© The Macmillan Press Ltd., 1983

route at a volume of $0.01\,ml\,g^{-1}$ 30 min before cytotoxic drug administration. Cytotoxic drugs were obtained, dissolved and administered as shown in Table I.

### Results

*Toxicity of WR 2721 alone*

In an earlier study we reported the acute $LD_{50}$ (7 days) of batch AJ 68.2 in female C3H mice to be $550\,mg\,kg^{-1}$. Two recent small experiments with this same batch have produced values of 650 and $550\,mg\,kg^{-1}$. In the second of these experiments batch AJ 68.4 was also tested and gave an identical value of $550\,mg\,kg^{-1}$.

We also found (Honess & Twentyman, unpublished) that whereas $200\,mg\,kg^{-1}$ of batch AJ 68.2 produced essentially no change in mouse body temperature, a dose of $400\,mg\,kg^{-1}$ produced a fall of 4–5°C at 1 h after administration with recovery by 4–6 h. A comparative study of batches AJ 68.2 and AJ 68.4 has now been made and the change in 1 h body temperature with dose of WR 2721 was similar for the 2 batches.

In view of the potential complications due to hypothermia produced by $400\,mg\,kg^{-1}$ of WR 2721 and our previous finding of marked tumour protection against CTX by this dose (Twentyman, 1981), many of the current experiments have used a WR 2721 dose of $200\,mg\,kg^{-1}$. A number of experiments have, however, also included the higher dose of $400\,mg\,kg^{-1}$.

*Acute lethality of cytotoxic drugs*

Results of experiments to determine the median

**Table I** Cytotoxic drugs studied

| *Drug* | *Supplier* | *Preparation* | *Administered volume** ($ml\,g^{-1}$) |
|---|---|---|---|
| Cyclophosphamide (CTX) | Ward Blenkinsop Ltd. | Dissolve in HBSS | 0.005–0.02 |
| Melphalan (MEL) | Chester Beatty Research Institute | Dissolve in acidified ethanol. Dilute 1:10 in propylene glycol/ $K_2HPO_4$ buffer, final pH 7.4 | 0.01 |
| 1-(2-chloroethyl) -3-cyclohexyl- 1-nitrosourea (CCNU) | U.S. National Cancer Institute | Dissolve in absolute ethanol. Dilute 1:20 in 0.5% carboxy-methyl cellulose/ HBSS | 0.005–0.05 |
| Chlorambucil (CHL) | Chester Beatty Research Institute | Dissolve in absolute ethanol. Dilute 1:10 in arachis oil B.P. | 0.01 |
| *Cis*-dichlorodi-ammineplatinum II (*cis*-P) | Mead Johnson Laboratories | Dissolve in HBSS | 0.005–0.04 |

*All drugs administered by the i.p. route.

lethal dose ($LD_{50}$) at 30 days for various cytotoxic drugs in the presence or absence of WR 2721 are shown in Table II. These data are based on results of experiments in which 6 groups of 5 mice were treated with graded doses of the cytotoxic drugs. For CHL, most deaths occurred within 48 h of drug administration. For CCNU and *cis*-P almost all deaths occurred 5–8 days after treatment. For CTX deaths began at Day 7 and continued throughout the 30–day observation period.

*White cell count*

The depression of peripheral white cell count at 3 days after CTX is shown in Figure 1. It may be seen that no significant change in the pattern was brought about by pretreatment with WR 2721 at either of the dose levels used. This conclusion was confirmed in a repeat experiment.

Similar experiments in which CCNU and *cis*-P were combined with either 200 or 400 mg kg$^{-1}$ of WR 2721 produced similar results to that for CTX (i.e. no difference in depression of white cell count at Day 3). For CCNU, white cell counts were followed for a further 7 days and recovery was not modified by WR 2721 treatment.

*Tumour response*

The effect of WR 2721 pretreatment on the anti-

**Table II** Effect of WR2721 on acute toxicity of cytotoxic drugs

| *Drug* | *WR 2721 Dose (mg kg$^{-1}$)* | *$LD_{50}$ − WR 2721 (mg kg$^{-1}$)* | *$LD_{50}$ + WR 2721 (mg kg$^{-1}$)* | *P.F.* | *Note* |
|---|---|---|---|---|---|
| CTX | 400 | 217 (190–244) | 313 (251–375) | 1.44 (1.16–1.79) | a |
| | 400 | 273 (215–331) | 334 (303–365) | 1.22 (0.98–1.51) | a,b |
| | 400 | 225 (200–250) | 232 (193–272) | 1.03 (0.85–1.22) | a |
| | 200 | 252 (218–285) | 374 (343–405) | 1.48 (1.28–1.72) | a |
| | 200 | 308 (266–357) | >450 | >1.46 | c,e |
| | 200 | 253 (233–274) | 224 (185–272) | 0.89 (0.70–1.13) | c |
| | 200 | 279 (254–305) | 327 (283–378) | 1.17 (0.99–1.39) | d |
| CCNU | | | | | |
| | 400 | 54.9 (49.1–62.9) | 46.7 (40.1–54.4) | 0.85 (0.71–1.01) | |
| | 200 | 49.4 (44.2–55.3) | 58.2 (44.0–52.8) | 1.18 (1.02–1.37) | |
| | 200 | 54.9 (49.1–61.4) | 54.7 (50.1–59.7) | 1.00 (0.86–1.15) | |
| CHL | 300 | 25.3 (23.8–26.9) | 30.2 (27.6–33.1) | 1.19 (1.07–1.32) | |
| *Cis*-P | 400 | 16.8 (13.5–20.9) | 25.3 (23.1–27.7) | 1.51 (1.28–1.78) | |
| | 400 | 12.0 ( 6.5–22.2) | 22.7 (20.1–25.7) | 1.89 (1.01–3.53) | f |
| | 400 | 12.0 ( 6.5–22.2) | 20.6 (18.4–23.1) | 1.72 (0.92–3.20) | f,g |
| | 400 | 10.7 ( 9.0–12.8) | 18.8 (17.1–20.6) | 1.76 (1.44–2.14) | g |
| | 200 | 16.8 (13.5–20.9) | 19.4 (16.9–22.4) | 1.15 (0.89–1.50) | |
| | 200 | 19.1 (13.8–26.4) | 20.3 (16.6–24.8) | 1.06 (0.77–1.47) | |

Values are $LD_{50}$ (95% confidence limits) at 30 days, computed using the GLIM program for probit analysis.

$$\text{P.F. } (=\text{protection factor}) = \frac{LD_{50}(+\text{WR } 2721)}{LD_{50}(-\text{WR } 2721)}$$

Notes a) As previously reported (Twentyman, 1981)
b) $LD_{50}$ at 100 days
c) See note d
d) Combined data for 2 preceding experiments (marked c)
e) Survival did not fall to 50% at highest CTX dose in presence of WR 2721
f) Determinations carried out within same experiment using different batches of WR 2721
g) WR 2721 batch AJ 68.4

**Figure 1** Effect of WR 2721 on change in peripheral white cell count in mice measured 3 days after various doses of CTX. ● CTX alone; ▲ WR 2721 (200 mg kg$^{-1}$)—30 min—CTX; ■ WR 2721 (400 mg kg$^{-1}$)—30 min—CTX; 5 mice/group. Error bars indicate ±2 s.e. of the mean.

tumour effectiveness of the various cytotoxic drugs is shown in Tables III and IV. It may be seen that in the RIF-1 tumour we confirm our previous finding of marked protection against CTX by 400 mg kg$^{-1}$ of WR 2721 (Twentyman, 1981). In addition we have now also looked at the effect of growing the tumour intradermally in the flank instead of intramuscularly in the leg. There was again a tendency for WR 2721 to reduce the CTX-induced growth delay but the differences in this experiment were not significant at the 95% confidence level. Also in the RIF-1 tumour, protection of the tumour against *cis*-P is brought about by WR 2721. The data shown in Table III and Figure 2 indicate that the main effect is at lower doses of *cis*-P, and that for a growth delay of 4 days, the protection factor is around 1.4.

The data shown in Table IV indicate that only very small protection factors are seen for WR 2721 in combination with CCNU, MEL or CHL in the KHT tumour. We do not believe that this is a tumour difference, since in our earlier study (Twentyman. 1981) a clear protection by WR 2721 of the KHT tumour against CTX was seen. With such small effects, the differences in Table IV between groups receiving the same cytotoxic drug treatment but with or without WR 2721 are not significant in single experiments. It is of note, however, that, of

**Table III** Effect of WR 2721 on tumour growth delay in RIF-1

| *Drug* | *Dose (mg kg$^{-1}$)* | *WR 2721 Dose* | *Growth delay (days) (2 s.e. limits)* | *Note* |
|---|---|---|---|---|
| CTX | 100 | 0 | 9.1 (7.8–10.4) | |
| „ | 100 | 400 | 5.7 (4.4–7.1) | |
| CTX | 50 | 0 | 2.1 (1.4–2.9) | a |
| „ | 100 | 0 | 6.4 (4.8–8.2) | a |
| „ | 100 | 200 | 5.1 (4.1–6.2) | a |
| „ | 100 | 400 | 4.7 (3.9–6.0) | a |
| *Cis*-P | 8 | 0 | 8.9 (8.1–10.1) | |
| „ | 8 | 200 | 6.8 (5.6–8.2) | |
| *Cis*-P | 4 | 0 | 3.5 (1.7–5.7) | b |
| „ | 8 | 0 | 5.9 (3.7–8.6) | b |
| „ | 12 | 0 | 14.0 (13.6–15.5) | b |
| „ | 4 | 400 | 0.9 (0.3–1.5) | b,c |
| „ | 8 | 400 | 4.5 (3.2–5.8) | b,c |
| „ | 12 | 400 | 10.3 (8.9–11.7) | b,c |

Notes a) Tumours grown intra-dermally in the flank
b) These data also shown in Figure 4
c) WR 2721 batch AJ 68.4

**Table IV** Effect of WR 2721 on tumour growth delay in KHT

| *Drug* | *Dose (mg kg$^{-1}$)* | *WR 2721 Dose (mg kg$^{-1}$)* | *Growth delay (days) (2 s.e. limits)* |
|---|---|---|---|
| CCNU | | | |
| | 10 | 0 | 4.8 (4.1–5.6) |
| | 20 | 0 | 13.8 (12.9–14.7) |
| | 30 | 0 | 19.1 (16.2–22.1) |
| | 20 | 200 | 12.8 (12.5–13.1) |
| | 30 | 200 | 17.8 (16.5–19.1) |
| | 30 | 400 | 16.8 (14.9–18.8) |
| | 20 | 0 | 15.1 (14.1–16.1) |
| | 20 | 200 | 15.1 (12.4–18.2) |
| MEL | 8 | 0 | 1.1 (0.6–1.5) |
| | 12 | 0 | 5.2 (3.1–8.0) |
| | 12 | 200 | 4.5 (3.4–6.2) |
| | 12 | 0 | 8.2 (7.3–9.1) |
| | 12 | 200 | 8.0 (7.0–9.2) |
| CHL | 7.5 | 0 | 1.6 (0.7–2.8) |
| | 15 | 0 | 8.7 (7.3–10.3) |
| | 15 | 200 | 6.6 (5.5–7.7) |
| | 15 | 0 | 9.6 (7.8–11.7) |
| | 15 | 200 | 8.0 (6.9–9.2) |

**Figure 2** Growth delay in the RIF-1 tumour with increasing dose of *cis*-P. ● *cis*-P alone; ○ WR 2721 ($400\,mg\,kg^{-1}$) at 30 min before *cis*-P.

the 7 experiments in which a direct comparison was possible, the groups receiving WR 2721 showed a smaller growth delay in 6 cases and the delays were identical in the seventh.

### Discussion

The original data on the action of WR 2721 as a differential radioprotector of normal tissues (summarised by Yuhas, 1980*a*) showed two notable features. Firstly, the protection of a variety of different normal tissues in the mouse and in the rat could be obtained, with protection factors frequently in excess of 2.0 (neural tissue, however, being a notable exception). Secondly, with only a rare exception, no protection of tumours was found. More recently, however, a number of papers have appeared which report significant radioprotection of a number of different murine solid tumours (Rojas *et al*., 1982; Clement & Johnson, 1982) and of micrometastases in the lungs of mice (Milas *et al*., 1982).

This same course of events has been followed with regard to data on the ability of WR 2721 to protect normal tissues selectively from the effects of cytotoxic drugs. Reports of protection of normal tissues against nitrogen mustard (Yuhas, 1979), *cis*-P (Yuhas & Culo, 1980) and CTX (Yuhas *et al*., 1980) were all accompanied by findings of a lack of protection of tumours. Using the bone marrow CFUs assay in $LAF_1$ mice, Wasserman *et al*., (1981) examined the ability of WR 2721 to protect against nitrogen mustard, CTX, BCNU, *cis*-P and 5-fluorouracil and obtained protection factors in the range 1.5 to 4.6. In a parallel study of the growth delay in EMT6 tumours little or no protection was seen (although it is not possible to estimate protection factors from the data). More recently, however, our own studies with CTX (Twentyman, 1981) and those of Clement & Johnson (1982) with MEL and CTX have shown significant protection. It must therefore be recognised that differential radio- and chemo-protection by WR 2721 are by no means absolute and that detailed studies of therapeutic ratios using a variety of normal tissue endpoints are required.

The dose of WR 2721 used and its time of administration with respect to radiation or cytotoxic drugs are important parameters to be considered in any study of interactions. In most radiation studies a time of 15–30 min has been chosen as this allows peak levels of drug to be attained in most normal tissues whilst allowing little time for the slower absorption into tumours (Yuhas, 1980*b*). A similar rationale has been used in chemoprotection experiments (Yuhas, 1979; Yuhas & Culo, 1980; Yuhas *et al*., 1980; Twentyman, 1981). A potential artefact of drug interaction in the bloodstream has, however, been pointed out when very short times (5–15 min for nitrogen mustard) are used (Yuhas, 1979).

Doses of WR 2721 used have generally been in the range of $200–600\,mg\,kg^{-1}$. In studies of radiation-induced haemopoietic death in 4 strains of mice, Yuhas (1980*a*) found that a protection factor of 2.0 was achieved at a WR 2721 dose of $200\,mg\,kg^{-1}$, compared with a value of 2.7 in the dose range $400–600\,mg\,kg^{-1}$. For radiation damage to the mouse jejunum, a plateau of protection was achieved at a WR 2721 dose of $200\,mg\,kg^{-1}$, although further protection was seen in the mouse testis by increasing the dose from $300–500\,mg\,kg^{-1}$ (Milas *et al*., 1982). Chemoprotection against nitrogen mustard (30-day survival) gave a protection factor of 1.5 for $200\,mg\,kg^{-1}$ of WR 2721 compared with 1.9–2.0 at $400\,mg\,kg^{-1}$, subsequently falling back to 1.5 at $500\,mg\,kg^{-1}$ (Yuhas, 1979). Protection against the renal toxicity of *cis*-P in the mouse (as measured by day-5 elevation of blood urea nitrogen) was by factors of 1.2 and 1.5 at WR 2721 doses of 100 and $200\,mg\,kg^{-1}$ respectively (Yuhas *et al*., 1980). In summary, therefore, although increases in protection factors are sometimes seen above a dose of $200\,mg\,kg^{-1}$ of WR 2721, the major component of protection is usually seen at this dose level with typical protection factors of around 1.5.

The data presented in this paper are, therefore, somewhat at variance with other reports on differential chemoprotection. In our earlier paper (Twentyman, 1981) we obtained a mean protection factor of 1.25 for $400\,mg\,kg^{-1}$ of WR 2721 with

CTX. A single experiment using 200 mg kg$^{-1}$, however, gave a higher protection factor of 1.48. Two further experiments, now reported in this paper give a combined value of 1.17 for 200 mg kg$^{-1}$, thus reducing the weight of the earlier relatively high value. For CCNU, the mean value at 200 mg kg$^{-1}$ is 1.1, whereas a single experiment at 400 mg kg$^{-1}$ gave no protection (factor = 0.85). Similarly for 300 mg kg$^{-1}$ of WR 2721 in conjunction with CHL a protection factor of 1.19 was obtained. For WR 2721 together with *cis*-P, a marked dose dependence was seen, values of 1.06 and 1.15 being obtained at 200 mg kg$^{-1}$ of WR 2721 but four values between 1.5 and 1.9 at 400 mg kg$^{-1}$.

Using depression of peripheral white cell count at Day 3 after drug treatment, however, we found no protection against CTX, CCNU, or *cis*-P at either 200 or 400 mg kg$^{-1}$ of WR 2721. This result, in particular, is in marked contrast with data of Wasserman *et al.*, (1981) where protection factors for mouse bone marrow CFUs of 2.4 and 3.2 were obtained for CTX and *cis*-P respectively (together with values of 4.6 and 1.5 for nitrogen mustard and BCNU). Clearly there are considerable differences in the target cell populations for the two assays. The 3-day nadir of white cell count is likely to reflect the effect of the drugs mainly upon the proliferating committed precursors of the white cell series with the subsequent recovery rate being determined by the earlier precursors (including CFUs). Although most progenitors of the granulocyte series are located in the bone marrow, lymphoid precursors are more widely distributed. As around 70% of peripheral white cells in the mouse are lymphocytes, drug effects in sites other than the marrow will be very important in determining the Day 3 peripheral count. Site-dependent differences in drug levels or in the degree of interaction between WR 2721 and the various cytotoxic drugs may, therefore, be involved in these conflicting results. In addition, it should be noted that the CFUs assays in the study of Wasserman *et al.*, were carried out at 2 h after drug administration. It is likely that a 5°C drop in mouse body temperature (as caused by 400 mg kg$^{-1}$ of WR 2721—see **Materials and methods**) will cause considerable changes in cytotoxic drug activation and metabolism and the use of such a short time of assay may be misleading if drug availability times are considerably prolonged. This problem does not, of course, arise when considering an *in situ* endpoint such as white cell count depression.

We have confirmed our earlier finding of tumour protection against CTX by WR 2721, being greater at 400 mg kg$^{-1}$ than at 200 mg kg$^{-1}$. We have also found protection against *cis*-P at 200 and 400 mg kg$^{-1}$ of WR 2721. For the other cytotoxic agents there appeared to be a trend towards tumour protection, but with very small protection factors.

In conclusion, therefore, our data for differential chemoprotection by WR 2721 are distinctly less encouraging than the balance of other data in the literature. In most of our $LD_{50}$ experiments, the protection factors were very small and in none of our white cell count experiments was significant protection seen. Only for *cis*-P in combination with 400 mg kg$^{-1}$ of WR 2721 was protection in excess of 1.5 seen for $LD_{50}$. For this drug (and for CCNU) almost all deaths in the $LD_{50/30}$ experiments occur 5–8 days after drug administration and are likely to be due to gastrointestinal damage. This $LD_{50}$ factor may therefore be indicative of considerable variations in protection factors between various critical tissues. As the tumour protection factor for this combination is $<1.2$ at *cis*-P doses in the $LD_{50}$ region, then differential chemoprotection may occur and thus this combination may be worthy of further study.

I thank Daryl Knight and Kate Smith for their technical assistance.

### References

CLEMENT, J.J. & JOHNSON, R.K. (1982). Influence of WR 2721 on the efficacy of radiotherapy and chemotherapy of murine tumours. *Int. J. Radiat. Oncol. Biol. Phys.*, **8**, 539.

KALLMAN, R.F., SILINI, G. & VAN PUTTEN, L.M. (1967). Factors influencing the quantitative estimation of the *in vivo* survival of cells from solid tumours. *J. Natl Cancer Inst.*, **39**, 539.

MILAS, L., HUNTER, N., REID, B.O. & THAMES, H.D. (1982). Protective effects of S-2-(3-aminopropylamino)-ethyl phosphorathioic acid against radiation damage of normal tissues and a fibrosarcoma in mice. *Cancer Res.*, **42**, 1888.

ROJAS, A., STEWART, F.A. & DENEKAMP, J. (1982). Experimental radiotherapy with WR 2721 and misonidazole. *Int. J. Radiat. Oncol. Biol. Phys.*, **8**, 527.

TWENTYMAN, P.R. (1981). Modification of tumour and host response to cyclophosphamide by misonidazole and by WR 2721. *Br. J. Cancer*, **43**, 745.

TWENTYMAN, P.R., KALLMAN, R.F. & BROWN, J.M. (1979). The effect of time between X-irradiation and chemotherapy on the growth of three solid mouse tumours: I. Adriamycin. *Int. J. Radiat. Oncol. Biol. Phys.*, **5**, 1255.

TWENTYMAN, P.R., BROWN, J.M., GRAY, J.W., FRANKO, A.J., SCOLES, M.A. & KALLMAN, R.F. (1980). A new mouse tumour model system (RIF-1) for comparison of end-point studies. *J. Natl Cancer Inst.*, **64**, 595.

WASSERMAN, T.H., PHILLIPS, T.L., ROSS, G. & KANE, J.L.(1981). Differential protection against cytotoxic chemotherapeutic effects on bone marrow CFU/s by WR 2721. *Cancer Clin. Trials*, **4**, 3.

YUHAS, J.M. (1979). Differential protection of normal and malignant tissues against the cytotoxic effects of mechlorethamine. *Cancer Treat Rep.*, **63**, 971.

YUHAS, J.M. (1980*a*). On the potential application of radio-protective drugs in radiotherapy. In *Radiation-Drug Interactions in Cancer Management*. (Ed. Sokol) New York: Wiley & Sons.

YUHAS, J.M. (1980*b*). Active versus passive absorption kinetics as the basis for selective protection of normal tissues by S-2-(3-aminopropylamino)-ethyl phosphorothioic acid. *Cancer Res.*, **40**, 1519.

YUHAS, J.M. & CULO, F. (1980). Selective inhibition of the nephrotoxicity of cis-dichlorodiammineplatinum by WR 2721 without altering its anti-tumour properties. *Cancer Treat Rep.*, **64**, 57.

YUHAS, J.M., SPELLMAN, J.M., JORDAN, S.W., PARDINI, M.C., AFZAL, S.M.J. & CULO, F. (1980). Treatment of tumours with the combination of WR 2721 and cis-dichlorodiammineplatinum (II) or cyclophosphamide. *Br. J. Cancer*, **42**, 574.